# Synthesis, Design, and Structure–Activity Relationship of the Pyrimidone Derivatives as Novel Selective Inhibitors of *Plasmodium falciparum* Dihydroorotate Dehydrogenase

**DOI:** 10.3390/molecules23061254

**Published:** 2018-05-24

**Authors:** Le Xu, Wenjie Li, Yanyan Diao, Hongxia Sun, Honglin Li, Lili Zhu, Hongchang Zhou, Zhenjiang Zhao

**Affiliations:** 1 Shanghai Key Laboratory of New Drug Design, School of Pharmacy, East China University of Science and Technology, Shanghai 200237, China; 13162212770@163.com (L.X.); wjli12345@126.com (W.L.); diaoyan1025@126.com (Y.D.); shx7982@163.com (H.S.); hlli@mail.shcnc.ac.cn (H.L.); 2 Department of Microbiology, Medical School of Huzhou Teachers College, Huzhou 313000, China

**Keywords:** *P. falciparum*, *Pf*DHODH, pyrimidone, antimalarial agents

## Abstract

The inhibition of *Plasmodium falciparum* dihydroorotate dehydrogenase (*Pf*DHODH) potentially represents a new treatment option for malaria, as *P. falciparum* relies entirely on a de novo pyrimidine biosynthetic pathway for survival. Herein, we report a series of pyrimidone derivatives as novel inhibitors of *Pf*DHODH. The most potent compound, **26**, showed high inhibition activity against *Pf*DHODH (IC_50_ = 23 nM), with >400-fold species selectivity over human dihydroorotate dehydrogenase (*h*DHODH). The brand-new inhibitor scaffold targeting *Pf*DHODH reported in this work may lead to the discovery of new antimalarial agents.

## 1. Introduction

Malaria is a mosquito-transmitted disease caused by protozoan parasites of the *Plasmodium* species [1,2]. The World Health Organization (WHO) estimated that there were nearly 216 million episodes of malaria and 445,000 deaths from malaria globally in 2016 [3]. Artemisinin combination therapies (ACTs) are the current first-line treatment for the deadliest form of malaria. However, the reduced efficacy of first-line treatments using artemisinin (compound **1**) [4] and its derivatives (associated with Kelch 13 propeller protein mutations 7-95430) is now prevalent at the Cambodia–Thailand border and poses a serious threat to malaria control programs globally [5,6,7,8]. Chloroquine (compound **2**) [9] and pyrimethamine (compound **3**) [10], used as the mainstay of antimalarial chemotherapy, have now compromised the development of resistance [11]. To tackle the problem of drug resistance, various strategies have been developed to treat malaria [12,13]. For instance, Gilbert’s group discovered that DDD107498 exhibits a novel spectrum of antimalarial activity against multiple life-cycle stages of the *Plasmodium* parasite [14]. 

Dihydroorotate dehydrogenase (DHODH) is a rate-limiting enzyme that is required for the fourth step of de novo pyrimidine biosynthesis, converting dihydroorotate (DHO) to orotate (ORO) with the participation of the cofactors flavin mononucleotide (FMN) and ubiquinone (CoQ) [15,16,17]. Pyrimidine-based biosynthesis represents a basic biological and physiological process that is crucial for RNA and DNA production and cell proliferation. The mammalian cells generate pyrimidines through both de novo and salvage pathways for survival, while plasmodium parasites lack the necessary genes for the former, resulting in de novo pyrimidine synthesis as the vital pathway for the parasite [18]. Therefore, *Pf*DHODH has been considered as the prospective target for the conquest of malaria [19,20,21]. DHODHs are divided into two families in the light of different amino acid sequences and coenzyme dependence. Human dihydroorotate dehydrogenase (*h*DHODH) and *Pf*DHODH belong to family II, located on the inner mitochondrial membrane, which utilizes ubiquinone as the terminal oxidant [22]. 

**Figure 1:**
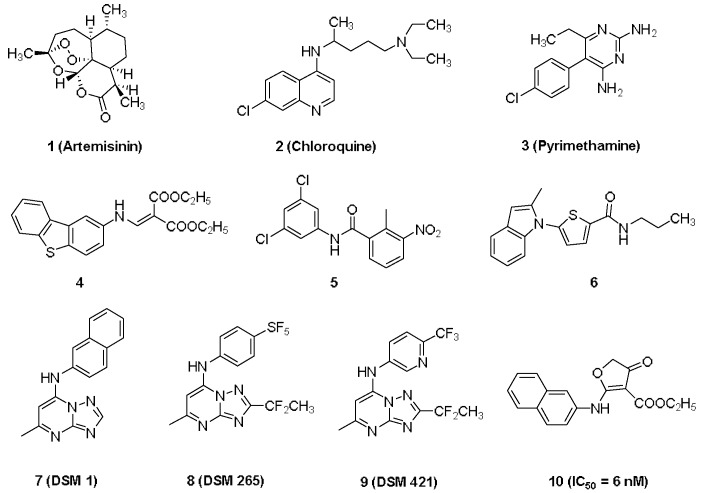
Structures of reported antimalarial agents **1**–**3**, *Pf*DHODH inhibitors **4**–**9**, and the lead compound **10** in this study.

Many *h*DHODH inhibitors have been corroborated to be effective against a number of diseases, such as cancer, rheumatoid arthritis (RA), psoriasis, and lupus erythematosus [23,24,25]. Some successes in identifying *Pf*DHODH inhibitors on the basis of the modification of reported *h*DHODH inhibitors (e.g., compound **4**) are well known [24]. Although sharing a highly conserved sequence in their C-terminal domains, *Pf*DHODH and *h*DHODH possess huge variation in terms of the amino acid sequence in the N-terminal domain, which facilitates further discovery of specific inhibitors of *Pf*DHODH [22]. In addition, many different kinds of selective and promising *Pf*DHODH inhibitors have been developed through target-based high-throughput screening (e.g., compounds **5**–**7**) (as shown in Figure 1) [26,27,28]. What is more, X-ray structures of *Pf*DHODH in complex with some representative compounds were used to develop more potent, non-cross-resistant selective antimalarial agents, leading to the identification of compound **8** (DSM 256), which has progressed in phase II clinical trials [29]. Furthermore, compound **9** (DSM 421) is a preclinical development candidate for the treatment of malaria, which has improved solubility, lower intrinsic clearance, and increased plasma exposure after oral dosing compared with DSM 265 [30]. 

In our previous work, we reported the identification of compound **10** as a *Pf*DHODH inhibitor by the structure-based virtual screening strategy integrating molecular Glide docking with Prime/MM-GBSA re-scoring on the basis of the complex crystal structure of compound **7** (DSM 1) with *Pf*DHODH (PDB code 3I65) [31]. Compound **10** selectively inhibited *Pf*DHODH (IC_50_ = 6 nM, with >14,000-fold species selectivity over *h*DHODH) and parasite growth in vitro (IC_50_ of 15 and 18 nM against 3D7 and Dd2 cells, respectively), but it was less effective in vivo as a result of its poor metabolic properties [31]. Recently, several pyrimidone derivatives based on compound **10** as a lead compound were obtained in this work and showed high selectivity over *h*DHODH, suggesting potential applications as novel antimalarial agents.

## 2. Results and Discussion

### 2.1. Proposed Binding Pose of Lead Compound **10** in the Ubiquinone Binding Pocket of PfDHODH

**Figure 2:**
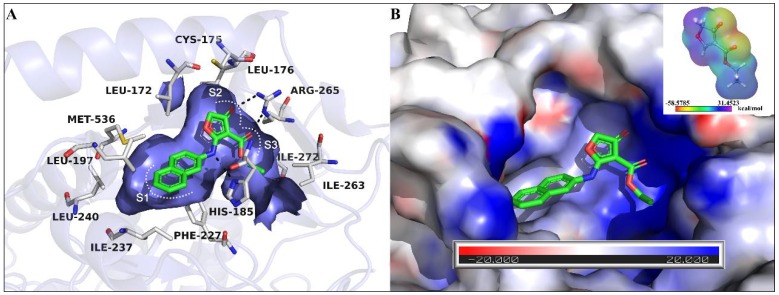
(**A**) Proposed binding pose of lead compound **10** in the ubiquinone binding pocket of *Pf*DHODH. Key residues around the binding pocket are shown as white lines and ligand is shown as green sticks. The hydrogen bonds are labeled as black dashes. (**B**) Electrostatic complementarity between the dihydrofuranone moiety of compound **10** and the binding site near Arg265. The electrostatic potential surface of *Pf*DHODH shown between −20 (red) and +20 kT/e (blue) was calculated using APBS tool [33,34,35]. The ESP of the dihydrofuranone fragment was calculated by Jaguar with DFT at B3LYP/cc-pVTZ(-f) level.

To thoroughly explore the binding mode of compound **10** in the ubiquinone binding pocket of *Pf*DHODH, flexible induced-fit docking [32] was implemented in Maestro v10.1 on the basis of the crystal structure of *Pf*DHODH in complex with DSM1 (PDB code 3I65). As shown in Figure 2A, the entrance of the ubiquinone binding channel is relatively closed in *Pf*DHODH; thus compound **10** is totally buried inside the protein. The naphthyl group sits in the region close to the entrance and fits the pocket well through hydrophobic interactions with adjacent residues such as Met536, Leu197, Leu240, Ile237, and Phe227. The ester group is located almost in the same plane of the dihydrofuranone moiety, with its carbonyl group pointing in the same direction as that on the dihydrofuranone ring. Both the carbonyl groups could form favorable hydrogen bond interactions with Arg265. Additionally, the binding pocket near Arg265 has a strongly positive electrostatic surface potential, which is complementary to the two carbonyl groups with negative electrostatic potential (the minimum ESP values of the two oxygen atoms are both −58.53 kcal/mol). An additional hydrogen bond is presumably formed between the bridging nitrogen atom and His185, which is considered to be essential for the activities and selectivities of *Pf*DHODH inhibitors. The ethyl group extends to the cavity near the FMN binding site, forming beneficial hydrophobic effects with residues Ile263 and Ile272 that could help to enhance the potency of compound **10**.

### 2.2. The Structural Optimization Strategy using the Lead Compound ***10***

**Figure 3:**
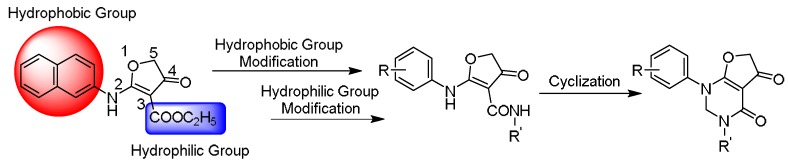
Diagram for the structural optimization strategy.

According to rational drug design and the proposed binding mode described above, the desired *Pf*DHODH inhibitor should embrace two functional parts, a hydrogen bond forming “head” linked via a nitrogen to a hydrophobic “tail”. In the hydrophilic pocket composed by subsites 2 and 3, the functional groups (e.g., carbonyl groups) could form hydrogen bonds with the guanidine group of Arg265, which is indispensable for maintaining the inhibitory activity against *Pf*DHODH. As for the hydrophobic pocket of subsite 1, the naphthyl ring is more suitable compared with the reported *Pf*DHODH inhibitors in terms of the size that the cavity can accommodate [31]. Consequently, with the attempt to discover more potent *Pf*DHODH inhibitors for further pharmacological study, as well as to verify the structure–activity relationship, an optimization strategy using the lead compound **10** was carried out through three modifications: (1) hydrophobic modification on the aromatic ring, (2) hydrophilic group modification on the dihydrofuranone ring, and (3) cyclization forming a new scaffold to enhance the stability (as shown in Figure 3).

### 2.3. Chemistry

**Scheme 1:**
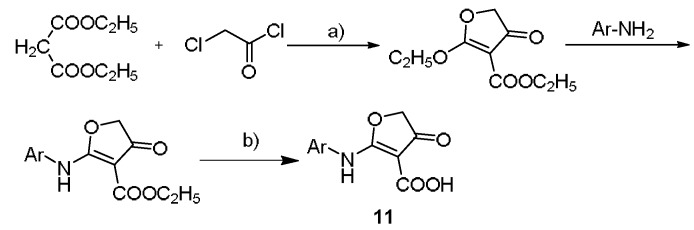
Reagents and conditions: (**a**) sodium hydride, diethyl malonate, 2-chloroacetyl chloride, and THF, at rt for 1 h, at 40–45 °C for 5h, and under reflux for 18 h; (**b**) LiOH–H_2_O, MeOH–H_2_O (55–60 °C) for 12 h.

**Scheme 2:**
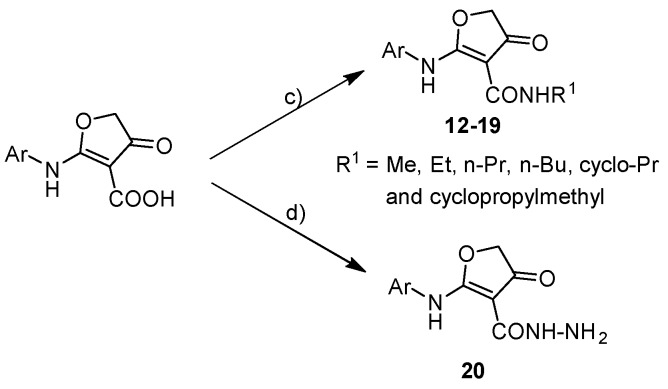
Reagents and conditions: (**c**) RNH_2_, HOBt, EDC, DIPEA, and CH_2_Cl_2_, overnight at rt; (**d**) 80% hydrazine hydrate and *N*,*N*′-carbonyldiimidazole (CDI), overnight at rt.

The lead compound **10**, with a dihydrofuranone core, was regarded as the well-designed structural foundation in our initial optimization. First, we replaced the easily metabolized ester group with an amide group. The preparation of the amide derivatives started with compound **11**, which served as an important intermediate (Scheme 1). To avoid byproducts during ester hydrolysis, the temperature must be controlled accurately [36]. With the purpose to further study the structure–activity relationships and improve the pharmacological properties, a series of dihydrofuranone derivatives were designed and synthesized (Scheme 1) [37]. Amides were synthesized in the presence of HOBt, EDCI, and DIPEA. Compound **20** was obtained by replacing alkylamines with 80% hydrazine hydrate and using N, N'-carbonyldiimidazole (CDI) in the routes depicted in Scheme 2.

**Scheme 3:**
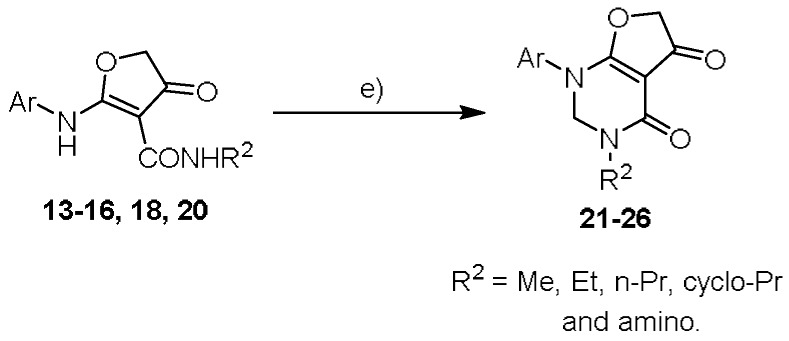
Reagents and conditions: (**e**) paraformaldehyde, NaOH and EtOH, at rt for 1 h and at 70 °C for another 8 h.

According to the predicted binding pose of the lead compound **10**, two carbonyl groups that orient in the same direction are necessary for the potent activity. Therefore, we used paraformaldehyde to form the rigid six-membered ring that could fix the two carbonyl group orientations well in space. For this reason, compounds **13**–**16**,**18** and **20** were subsequently transformed to the pyrimidone derivatives **21**–**26** by cyclization (Scheme 3) [38]. All structures of the final products were determined by spectroscopic techniques.

### 2.4. Inhibitory Activities against PfDHODH and SAR Study

#### 2.4.1. Hydrophobic Group Modification

Because the *Pf*DHODH inhibition potency was particularly sensitive to the substitution pattern at the phenyl ring [39], an optimization strategy was carried out: modification on the hydrophobic moiety substituted by phenyl (compound **12**) and 5-aminoindan (compound **13**). Compared with compounds **12** and **13**, compound **15** containing a 2-naphthyl moiety was more active against *Pf*DHODH, with an IC_50_ value of 4.8 µM. Notably, the phenyl ring as the hydrophobic group reversed the species selectivity of compound **12**, which showed an IC_50_ value of 2.4 µM against *h*DHODH and was inactive against *Pf*DHODH. The above results validate that using 2-naphthyl as the hydrophobic group is beneficial to keep a high potency against *Pf*DHODH (as shown in Table 1).

#### 2.4.2. Hydrophilic Group Modification

On the basis of previous results using 2-naphthyl as the hydrophobic group (R1 = 2-naphthyl), compounds **11** and **14**–**20** were obtained. In this section, the emphasis is on evaluating the effect of the structural changes of the hydrophilic group on the inhibitory activity against *Pf*DHODH. In the absence of an ethoxycarbonyl group on the dihydrofuranone scaffold, compound **11** displayed lower inhibitory activity in comparison with **10**, which suggests that it would be strongly disfavored if the derivatives do not possess any substituent at the 3-position of the scaffold (as shown in Figure 3). Next, derivatives with different amide groups (compounds **12**–**20)** were synthesized to identify the optimal alkyl chain that could fit the binding cavity. Amides with different chain lengths were introduced to investigate their effects on the inhibitory activities. Compared with compound **15** (IC_50_ = 4.8 µM), compound **14** (IC_50_ = 9 µM) showed a decline in the inhibitory activity against *Pf*DHODH; the reason may be attributed to the shorter alkyl chain, which mismatched the size of the cavity. However, compounds **16** and **17** lost their activities because of the long alkyl chains, which may have resulted in collisions with residues such as Ile263 and Ile272. Compound **18** inhibited *Pf*DHODH with an IC_50_ value of 6 µM, which indicated that the size of the cyclopropyl was suitable for the hydrophobic cavity to some extent. However, compound **19** with cyclopropanemethylamine lost the inhibitory activity against *Pf*DHODH owing to the increased size of the alkyl side chain. According to our present study, ethyl and cyclopropyl are well matched with the size and shape of the cavity. Compound **20** equipped with the amino displayed an IC_50_ value of 70 nM, likely a result of forming a hydrogen bond interaction with the adjacent residue His185. Therefore, a suitable substituent group in the 3-position and the amino group forming the hydrogen bonding interaction with His185 in *Pf*DHODH played a vital role for potent activities of *Pf*DHODH inhibitors (as shown in Table 1).

#### 2.4.3. Cyclization Forming a New Scaffold

**Figure 4:**
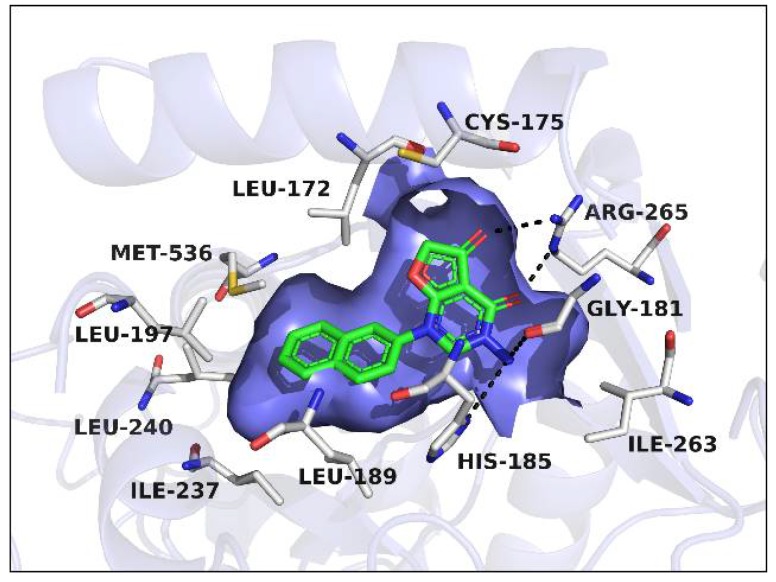
Proposed binding pose of compound **26** in the ubiquinone binding pocket of *Pf*DHODH. Key residues around the binding pocket are shown as white lines, and ligand is shown as green sticks. The hydrogen bonds are labeled as black dashes.

After cyclization forming a new scaffold, a six-membered ring could stabilize two carbonyl groups orientated in the same direction, which formed hydrogen bonds with Arg265 of *Pf*DHODH. It is clear that compound **22**–**26** showed higher inhibitory activities compared with the corresponding uncyclized amide mimics (Table 1). The reason for all these compounds with the new scaffold presenting high inhibitory activities against *Pf*DHODH can be elucidated from the predicted binding pose in our work. The predicted binding pose of compound **10** revealed that the bridge nitrogen atom not only linked the hydrophilic group to the hydrophobic group, but also interacted with His185 via a hydrogen bond, which is very important for the inhibitory activity. We designed new *Pf*DHODH inhibitors that contained a new hydrogen bond donor interacting with His185. In our present study, compound **26** was the most potent candidate against *Pf*DHODH, which had an IC_50_ value equal to 23 nM and was 3-fold more active than compound **20**. The predicted binding pose of compound **26** is shown in Figure 4. Two carbonyl groups formed hydrogen bonds with residues Arg265, and the nitrogen atom in the amino group formed an additional hydrogen bond with His185. Briefly, cyclization enhanced the structural stability and maintained the hydrogen bond interactions between the two carbonyl groups and the key residue Arg265.

## 3. Materials and Methods

All chemical reagents and solvents were obtained from commercial sources and were used without further purification. Melting points (Mp’s) were recorded on a WRS-1B-digital melting point apparatus and are uncorrected. High-resolution electron mass spectra (ESI-TOF) were produced using a Micromass LC-TOF spectrometer (Waters, Milford, MA, US). ^1^H- and ^13^C-NMR spectra were recorded on a Bruker AM-400 (^1^H at 400 MHz; ^13^C at 100 MHz) spectrometer (Bruker, Fallanden, Switzerland)) with DMSO-*d*_6_ or CDCl_3_ as the solvent and TMS as the internal standard. Chemical shifts are reported in δ (parts per million). Analytical thin-layer chromatography (TLC) was performed on precoated plates (silica gel 60 F254), and spots were visualized with ultraviolet (UV) light. The following are used to explain the multiplicities: s = singlet, d = doublet, t = triplet, q = quartet, m = multiplet, coupling constant (Hz), and integration. 

### 3.1. In Vitro Enzyme Assay 

Protein expression, purification, and enzyme activity determination of *Pf*DHODH (Phe158–Ser569) and *h*DHODH (Met30–Arg396) were performed according to our previous work [31]. The enzymes were diluted to a final concentration of 10 nM using the assay buffer containing 50 mM HEPES (pH 8.0), 150 mM KCl, 0.1% Triton X-100 supplemented with 100 µM UQ_0_, and 120 µM DCIP. The mixture was transferred into a 96-well plate and incubated with or without various amounts of inhibitors for 5 min at room temperature, and then the dihydroorotate was added to a final concentration of 500 µM to initiate the reaction. The reaction was monitored by measuring the decrease in DCIP in the absorption at 600 nm at each 30 s over a period of 6 min. Brequinar and DSM1 were measured as the positive controls for *h*DHODH and *Pf*DHODH, respectively. Percent inhibition relative to no inhibitor control was calculated by (1 − V_i_/V_0_) × 100. For the determination of the IC_50_ value of the inhibitor, eight or nine different concentrations were applied. Each inhibitor concentration point was tested in triplicate, and the IC_50_ values were calculated using the sigmoidal fitting option of the program Origin 8.0 (Originlab, Northampton, MA, US).

### 3.2. Chemistry Experiment

#### 3.2.1. General Procedure for the Synthesis of Intermediates

##### Synthesis of the ethyl 2-(substituted arylamino)-4-oxo-4,5-dihydrofuran-3 carboxylate

Diethyl malonate (63 mmol) dissolved in dry THF (15 mL) was added to a stirred solution of sodium hydride (71 mmol, 60%) in dry THF (10 mL), and the mixture was stirred for 20 min at 0–10 °C. Then the reaction mixture was stirred at room temperature for 10 min, and 2-chloroacetyl chloride (32 mmol) in dry THF (15 mL) was added. The solution was kept at room temperature for 1 h and at 40–45 °C for another 5 h, and substituted aniline (11 mmol) in dry THF (100 mL) was added dropwise over 20 min. The reaction mixture was stirred at room temperature overnight and then heated under reflux for 18 h. After excess THF was evaporated off, the resulting residue was extracted with EtOAc (3 × 10 mL) and washed with brine (3 × 10 mL). The organic layer was dried (Na_2_SO_4_), concentrated under reduced pressure, and purified by column chromatography (PE: 1:1 *v*/*v* EtOAc) with 30–35% yield as a white solid.

##### Synthesis of the 2-(substituted arylamino)-4-oxo-4,5 dihydrofuranone-3-carboxylic acid

LiOH-H_2_O (10 mmol) was slowly added to a solution of ethyl 2-(substituted arylamino)-4-oxo-4,5-dihydrofuran-3-carboxylate (2 mmol) in MeOH–H_2_O (18 mL, 5:1 *v*/*v* MeOH/H_2_O) at 0 °C over 30 min. The reaction mixture was allowed to warm to 55–60 °C for 12 h with stirring. After MeOH was evaporated off, the aqueous residual was acidified to pH 1–2 with 1 N HCl and precipitated solid was filtered, washed with water, and dried under vacuum with 70–80% yield as a yellow solid.

##### Synthesis of compound **11**

LiOH–H_2_O (10 mmol) was slowly added to a solution of ethyl 2-(naphthalen-2-ylamino)-4-oxo-4,5-dihydrofuran-3-carboxylate (2 mmol) in MeOH–H_2_O (18 mL, 5:1 *v*/*v* MeOH/H_2_O) at 0 °C over 15 min. The reaction mixture was allowed to warm to 55–60 °C for 12 h with stirring. After MeOH was evaporated off, the aqueous residual was acidified to pH 1–2 with 1 N HCl and precipitated solid was filtered, washed with water, and dried under vacuum with 70–80% yield as a yellow solid. 
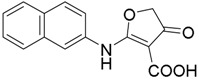


*2-(Naphthalen-2-ylamino)-4-oxo-4,5-dihydrofuran-3-carboxylic acid* (**11**); Mp: 164.4–165.0 °C. ^1^H-NMR (400 MHz, DMSO-*d*_6_): *δ* 11.47 (s, 1H), 10.55 (s, 1H), 8.08–7.89 (m, 4H), 7.62–7.40 (m, 3H), 4.07 (s, 2H). ^13^C-NMR (100 MHz, DMSO-*d*_6_): *δ* 197.3, 183.3, 165.3, 135.1, 133.2, 132.5, 130.0, 128.4, 128.2, 127.6, 127.4, 123.7, 123.4, 98.6, 38.7. HRMS (ESI): [M + H]^+^ calcd for C_15_H_11_NO_4_, 270.0688; found, 270.0688.

#### 3.2.2. General Procedure for Target Compounds **12**–**19**

HOBt (1.1 mmol), EDC (1.1 mmol), and DIPEA (1 mmol) were added to a solution of amine (1 mmol) and 2-(substituted amino)-4-oxo-4,5 dihydrofuranone-3-carboxylic acid (1 mmol) in dry DCM (5 mL) at 0 °C. The reaction mixture was stirred overnight at room temperature and then washed with 5% aqueous HCl (2 × 15 mL), 5% aqueous NaHCO_3_ (2 × 15 mL), and brine (2 × 15 mL) and was dried (Na_2_SO_4_) and concentrated under reduced pressure with purification by column chromatography (PE: 6:1, *v*/*v* EtOAc) with 20–25% yield as a white solid. 
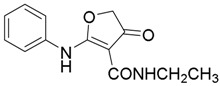


*N-Ethyl-4-oxo-2-(phenylamino)-4,5-dihydrofuran-3-carboxamide* (**12**); Mp: 146.9–147.4 °C. ^1^H-NMR (400 MHz, CDCl_3_): *δ* 11.51 (s, 1H), 7.47 (t, *J* = 8.0 Hz, 2H), 7.37 (d, *J* = 8.0 Hz, 2H), 7.35 (s, 1H), 4.39 (q, *J* = 7.2 Hz, 2H), 3.67 (s, 2H), 1.42 (t, *J* = 7.2 Hz, 3H). ^13^C-NMR (100 MHz, DMSO-*d*_6_): δ 191.0, 183.3, 165.5, 137.8, 130.0, 128.3, 125.6, 97.4, 59.7, 38.4, 14.9. HRMS (ESI): [M + H]^+^ calcd for C_13_H_14_N_2_O_3_, 247.1004; found, 247.1009. 
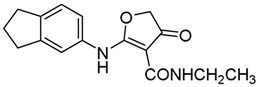


*2-[(2,3-Dihydro-1H-inden-5-yl)amino]-N-ethyl-4-oxo-4,5-dihydrofuran-3-carboxamide* (**13**); Mp: 127.7–128.2 °C. ^1^H-NMR (400 MHz, DMSO-*d*_6_): *δ* 11.08 (s, 1H), 7.32 (d, *J* = 8.0 Hz, 1H), 7.27 (s, 1H), 7.16 (d, *J* = 8.0 Hz, 1H), 4.22 (q, *J* = 7.2 Hz, 2H), 3.65 (s, 2H), 2.89 (t, *J* = 7.6 Hz, 4H), 2.09-2.02 (m, 2H), 1.26 (t, *J* = 7.2 Hz, 3H). ^13^C-NMR (100 MHz, DMSO-*d*_6_): *δ* 190.9, 183.5, 165.6, 145.7, 144.2, 135.8, 125.4, 123.6, 121.6, 97.1, 59.7, 38.4, 32.8, 32.4, 25.7, 14.9. HRMS (ESI): [M + H]^+^ calcd for C_16_H_18_N_2_O_3_, 287.1317; found, 287.1320. 
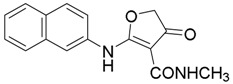


*N-Methyl-2-(naphthalen-2-ylamino)-4-oxo-4,5-dihydrofuran-3-carboxamide* (**14**); Mp: 126.3–126.9 °C. ^1^H-NMR (400 MHz, CDCl_3_): *δ* 11.65 (s, 1H), 7.67–7.79 (m, 4H), 7.42–7.51 (m, 3H), 4.62 (s, 2H), 3.32 (s, 3H). ^13^C-NMR (100 MHz, CDCl_3_): *δ* 192.4, 181.3, 165.9, 133.6, 134.9, 133.4, 131.8, 129.8, 127.8, 127.2, 126.5, 121.8, 120.6, 99.9, 78.3, 35.7. HRMS (ESI): [M + H]^+^ calcd for C_18_H_14_N_2_O_3_, 283.1004; found, 283.1011. 
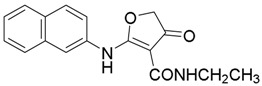


*N-Ethyl-2-(naphthalen-2-ylamino)-4-oxo-4,5-dihydrofuran-3-carboxamide* (**15**); Mp: 121.8–123.0 °C. ^1^H-NMR (400 MHz, CDCl_3_): *δ* 11.05 (s, 1H), 7.88–7.79 (m, 4H), 7.53–7.44 (m, 3H), 4.78 (s, 2H), 3.40−3.47 (m, 2H), 1.24 (t, *J* = 7.2 Hz, 3H). ^13^C-NMR (100 MHz, CDCl_3_): *δ* 190.5, 176.5, 164.3, 133.6, 132.7, 131.1, 129.5, 127.7, 127.6, 127.0, 125.9, 120.2, 118.1, 89.5, 75.7, 33.4, 15.1. HRMS (ESI): [M + H]^+^ calcd for C_17_H_16_N_2_O_3_, 297.1239; found, 297.1241. 
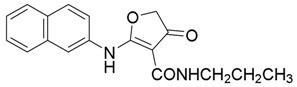


*2-(Naphthalen-2-ylamino)-4-oxo-N-propyl-4,5-dihydrofuran-3-carboxamide* (**16**); Mp: 126.2–127.2 °C. ^1^H-NMR (400 MHz, CDCl_3_): *δ* 12.95 (s, 1H), 8.91 (s, 1H), 7.92–7.84 (m, 4H), 7.56–7.29 (m, 3H), 3.75 (s, 2H), 3.37 (q, *J* = 6.4 Hz, 2H), 1.69-1.63 (m, 2H), 1.02 (t, *J* = 7.2 Hz, 3H). ^13^C-NMR (100 MHz, CDCl_3_): *δ* 193.8, 181.3, 165.9, 135.0, 133.4, 131.8, 129.8, 127.8, 127.2, 126.5, 121.8, 120.6, 99.6, 40.1, 38.5, 23.0, 11.5. HRMS (ESI): [M + H]^+^ calcd for C_18_H_18_N_2_O_3_, 333.1317; found, 333.1311. 
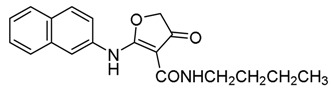


*2-(Naphthalen-2-ylamino)-4-oxo-N-propyl-4,5-dihydrofuran-3-carboxamide* (**17**); Mp: 140.1–141.0 °C. ^1^H-NMR (400 MHz, CDCl_3_): *δ* 12.94 (s, 1H), 8.89 (s, 1H), 7.93–7.84 (m, 4H), 7.58–7.44 (m, 3H), 3.75 (s, 2H), 3.41 (q, *J* = 6.8 Hz, 2H), 1.66-1.59 (m, 2H), 1.50-1.41 (m, 2H), 0.99 (t, *J* = 7.2 Hz, 3H). ^13^C-NMR (100 MHz, CDCl_3_): *δ* 193.8, 181.3, 165.9, 135.0, 133.4, 131.9, 129.8, 127.8, 127.2, 126.5, 121.8, 120.6, 99.6, 38.5, 38.1, 31.8, 20.2, 13.8. HRMS (ESI): [M + Na]^+^ calcd for C_19_H_20_N_2_O_3_, 347.1474; found, 347.1470. 
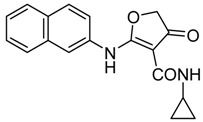


*N-Cyclopropyl-2-(naphthalen-2-ylamino)-4-oxo-4,5-dihydrofuran-3-carboxamide* (**18**); Mp: 140.8–41.6 °C. ^1^H-NMR (400 MHz, DMSO-*d*_6_): *δ* 12.62 (s, 1H), 8.78 (d, *J* = 3.6 Hz, 1H), 8.08–7.93 (m, 4H), 7.69–7.54 (m, 3H), 3.89 (s, 2H), 2.82–2.77 (m, 1H), 0.78–0.73 (m, 2H), 0.56–0.50 (m, 2H). ^13^C-NMR (100 MHz, CDCl_3_): *δ* 193.7, 181.2, 167.5, 134.9, 133.4, 131.8, 129.9, 127.9, 127.2, 126.5, 121.7, 120.5, 99.4, 38.6, 29.7, 21.6, 6.5. HRMS (ESI): [M + H]^+^ calcd for C_18_H_16_N_2_O_3_, 309.1161; found, 309.1165. 
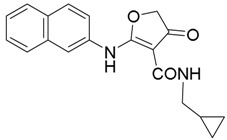


*N-(Cyclopropylmethyl)-2-(naphthalen-2-ylamino)-4-oxo-4,5-dihydrofuran-3-carboxamide* (**19**); Mp: 164.8–165.3 °C. ^1^H-NMR (400 MHz, DMSO-*d*_6_): *δ* 12.68 (s, 1H), 8.91 (t, *J* = 5.6 Hz, 1H), 8.06–7.97 (m, 4H), 7.62–7.55 (m, 3H), 3.92 (s, 2H), 3.19 (t, *J* = 6.4 Hz, 2H), 1.06-0.99 (m, 1H), 0.50–0.45 (m, 2H), 0.26–0.22 (m, 2H). ^13^C-NMR (100 MHz, CDCl_3_): *δ* 193.8, 181.3, 165.8, 134.9, 133.4, 131.8, 129.8, 127.9, 127.2, 126.5, 121.8, 120.6, 99.5, 43.0, 38.5, 10.9, 3.5. HRMS (ESI): [M + H]^+^ calcd for C_19_H_18_N_2_O_3_, 323.1317; found, 323.1315.

##### Synthesis of Compound **20**

CDI (1 mmol) was slowly added to a solution of ethyl 2-(naphthalen-2-ylamino)-4-oxo-4,5-dihydrofuran-3-carboxylic acid (1 mmol) in dry DCM (6 mL) at 0 °C for 2 h with stirring. Then to the reaction mixture was added 80% hydrazine hydrate overnight with stirring at room temperature. After the reaction, the precipitation was filtered, dried, and purified by column chromatography (EA: 10:1, *v*/*v* MeOH) in 23–27% in yield as a brown solid.

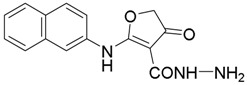


*2-(Naphthalen-2-ylamino)-4-oxo-4,5-dihydrofuran-3-carbohydrazide* (**20**); Mp: 167.3–167.9 °C. ^1^H-NMR (400 MHz, CDCl_3_): *δ* 12.11 (s, 1H), 8.02-7.91 (m, 4H), 7.66–7.51 (m, 3H), 5.58 (s, 2H), 5.19 (s, 2H). ^13^C-NMR (100 MHz, CDCl_3_): *δ* 194.7, 185.4, 162.7, 133.5, 132.6, 131.5, 132.2, 128.4, 128.2, 127.5, 127.2, 124.2, 120.3, 79.9, 76.2. HRMS (ESI): [M + H]^+^ calcd for C_15_H_13_N_3_O_3_, 284.0957; found, 284.0954.

#### 3.2.3. General Procedure for Target Compounds **21–26**

NaOH (1.5 mmol) was slowly added to a solution of N-substituted-2-(substituted amino)-4-oxo-4,5-dihydrofuran-3-carboxamide (1 mmol) in EtOH (6 mL) at 0 °C with stirring. The reaction mixture was allowed to warm to 45 °C for 1 h with stirring, and then paraformaldehyde (POM) (1.2 mmol) was added for 8 h at 70 °C. After the resulting mixture was filtered, the filtrate was concentrated under reduced pressure and purified by column chromatography (PE: 3:1 *v*/*v* EA) with 13.2–19.7% yield as a white solid. 
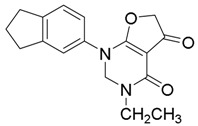


*1-(2,3-Dihydro-1H-inden-5-yl)-3-ethyl-2,3-dihydrofuro[2,3-d]pyrimidine-4,5(1H,6H)-dione* (**21**); Mp: 128.6–129.4 °C. ^1^H-NMR (400 MHz, CDCl_3_): *δ* 7.32 (d, *J* = 8.0 Hz, 1H), 7.27 (s, 1H), 7.16 (d, *J* = 8.0 Hz, 1H), 4.22 (q, *J* = 7.2 Hz, 2H),4.17 (s, 2H), 3.65 (s, 2H), 2.89 (t, *J* = 7.6 Hz, 4H), 2.09–2.02 (m, 2H), 1.26 (t, *J* = 7.2 Hz, 3H). ^13^C-NMR (100 MHz, CDCl_3_): *δ* 190.9, 183.5, 165.6, 145.7, 144.2, 135.8, 125.4, 123.6, 121.6, 97.1, 88.3, 59.7, 38.4, 32.8, 32.4, 25.7, 14.9. HRMS (ESI): [M + H]^+^ calcd for C_17_H_18_N_2_O_3_, 299.1317; found, 299.1325. 
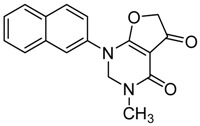


*3-Methyl-1-(naphthalen-2-yl)-2,3-dihydrofuro[2,3-d]pyrimidine-4,5(1H,6H)-dione* (**22**); Mp: 127.9–128.5 °C. ^1^H-NMR (400 MHz, CDCl_3_): *δ* 8.11–7.90 (m, 4H), 7.51–7.33 (m, 3H), 5.61 (s, 2H), 4.73 (s, 2H), 3.27 (s, 3H). ^13^C-NMR (100 MHz, CDCl_3_): *δ* 191.1, 183.4, 165.5, 135.4, 133.3, 132.3, 129.8, 128.4, 128.2, 127.5, 127.2, 124.2, 123.6, 97.6, 86.3, 77.8, 38.5. HRMS (ESI): [M + H]^+^ calcd for C_17_H_14_N_2_O_3_, 295.1004; found, 295.1011. 
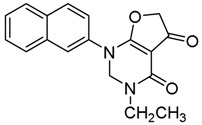


*3-Ethyl-1-(naphthalen-2-yl)-2,3-dihydrofuro[2,3-d]pyrimidine-4,5(1H,6H)-dione* (**23**); Mp: 128.3–129.0 °C. ^1^H-NMR (400 MHz, CDCl_3_): *δ* 8.11–7.90 (m, 4H), 7.51–7.33 (m, 3H), 5.61 (s, 2H), 4.73 (s, 2H), 3.27 (s, 3H). ^13^C-NMR (100 MHz, CDCl_3_): *δ* 191.1, 183.4, 165.5, 135.4, 133.3, 132.3, 129.8, 128.4, 128.2, 127.5, 127.2, 124.2, 123.6, 97.6, 86.3, 77.8, 38.5. HRMS (ESI): [M + H]^+^ calcd for C_17_H_14_N_2_O_3_, 309.1161; found, 309.1158. 
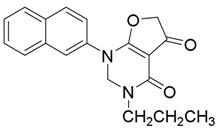


*1-(Naphthalen-2-yl)-3-propyl-2,3-dihydrofuro[2,3-d]pyrimidine-4,5(1H,6H)-dione* (**24**); Mp: 128.6–129.7 °C. ^1^H-NMR (400 MHz, DMSO-*d_6_*): *δ* 7.92-7.84 (m, 4H), 7.56–7.29 (m, 3H), 4.51 (s, 2H), 3.57 (s, 2H), 3.05 (q, *J* = 6.4, 2H), 1.69–1.63 (m, 2H), 1.33 (t, *J* = 7.2, 3H). ^13^C-NMR (100 MHz, DMSO-*d_6_*): *δ* 193.6, 182.4, 162.3, 135.4, 133.3, 132.3, 131.8, 128.4, 128.2, 127.5, 127.2, 124.2, 123.6, 97.6, 38.6, 38.5, 14.9. HRMS (ESI): [M + H]^+^ calcd for C_17_H_18_N_2_O_3_, 345.1317; found, 345.1314. 
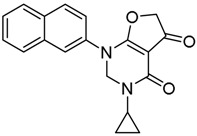


*3-Cyclopropyl-1-(naphthalen-2-yl)-2,3-dihydrofuro[2,3-d]pyrimidine-4,5(1H,6H)-dione* (**25**); Mp: 128.8–129.5 °C. ^1^H-NMR (400 MHz, DMSO-*d_6_*): δ 8.08–7.93 (m, 4H), 7.69–7.54 (m, 3H), 3.89 (s, 2H), 3.64 (s, 2H), 2.82–2.77 (m, 1H), 0.78-0.73 (m, 2H), 0.56–0.50 (m, 2H). ^13^C-NMR (100 MHz, CDCl_3_): *δ* 193.7, 181.2, 167.5, 134.9, 133.4, 131.8, 129.9, 127.9, 127.2, 126.5, 121.7, 123.5, 120.5, 99.4, 83.6, 77.4, 29.7, 21.6, 6.5. HRMS (ESI): [M + H]^+^ calcd for C_19_H_16_N_2_O_3_, 321.1161; found, 321.1152. 
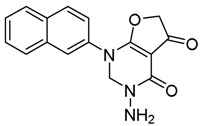


*3-Amino-1-(naphthalen-2-yl)-2,3-dihydrofuro[2,3-d]pyrimidine-4,5(1H,6H)-dione* (**26**); Mp: 168.4–169.2 °C. ^1^H-NMR (400 MHz, CDCl_3_): *δ* 10.33 (s, 2H), 7.83–7.67 (m, 4H), 7.65–7.43 (m, 3H), 5.51 (s, 2H), 4.88 (s, 2H).^13^C-NMR (100 MHz, CDCl_3_): *δ* 194.7, 185.4, 162.7, 133.5, 132.6, 131.5, 132.2, 128.4, 128.2, 127.5, 127.2, 124.2, 120.3, 87.3, 79.9, 76.2. HRMS (ESI): [M + H]^+^ calcd for C_16_H_13_N_3_O_3_, 296.0957; found, 296.0957.

## 4. Conclusions

In this work, a series of brand-new scaffold *Pf*DHODH-specific-inhibitor pyrimidone derivatives were obtained from docking analyses and structural optimizations. The most potent inhibitor **26** showed excellent inhibitory activity against *Pf*DHODH (IC_50_ = 23 nM) and high species selectivity over *h*DHODH. Through SAR studies, three preferential structural fragments were essential for these novel potent *Pf*DHODH inhibitors: (1) bicyclic systems such as “naphthyl-like” substituents as the hydrophobic group; (2) two carbonyl groups in the dihydrofuranone ring oriented in the same direction and that formed hydrogen bonds with the polar residue (Arg265); (3) a hydrogen bond donor (NH_2_ in compounds **20** and **26**) that was important for the inhibitory activity to interact with the imidazole group of His185. The results might be valuable for the novel scaffold *Pf*DHODH inhibitors to be developed as new antimalarial agents.

## Figures and Tables

**Table 1:** Structure and activity profiles of compounds **11**–**26** against *Pf*DHODH and human DHODH (*h*DHODH) in enzyme assays.

		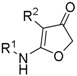	IC_50_ (μM)	
Compound	R^1^	R^2^	*Pf*DHODH *^a^*	*h*DHODH *^b^*
**11**	2-Naphthyl	COOH	0.106 ± 0.009	>10
**12**	Phenyl	CONHCH_2_CH_3_	>10	2.4 ± 0.1
**13**	5-Aminoindan	CONHCH_2_CH_3_	>10	>10
**14**	2-Naphthyl	CONHCH_3_	9 ± 1	>10
**15**	2-Naphthyl	CONHCH_2_CH_3_	4.8 ± 0.4	>10
**16**	2-Naphthyl	CONHCH_2_CH_2_CH_3_	>10	>10
**17**	2-Naphthyl	CONHCH_2_CH_2_CH_2_CH_3_	>10	>10
**18**	2-Naphthyl	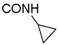	6 ± 1	>10
**19**	2-Naphthyl	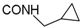	>10	>10
**20**	2-Naphthyl	CONH–NH_2_	0.07 ± 0.01	>10
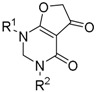				
**21**	5-Aminoindan	CH_2_CH_3_	>10	>10
**22**	2-Naphthyl	CH_3_	8 ± 1	>10
**23**	2-Naphthyl	CH_2_CH_3_	4 ± 1	>10
**24**	2-Naphthyl	CH_2_CH_2_CH_3_	12 ± 6	>10
**25**	2-Naphthyl	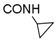	5 ± 1	>10
**26**	2-Naphthyl	NH_2_	0.023 ± 0.001	>10
DSM1	—	—	0.042 ± 0.004	—

^a^ The IC_50_ values of the compounds against *Pf*DHODH, in vitro assay, μM. ^b^ The IC_50_ values of the compounds against *h*DHODH, in vitro assay, μM.
